# Inhibition of X52 Corrosion in CO_2_-Saturated Brine by a Dialkyl-Diamide from Coffee Bagasse Oil

**DOI:** 10.3390/molecules28020763

**Published:** 2023-01-12

**Authors:** N. B. Gomez-Guzman, Jorge Canto, L. M. Martinez-de-la-Escalera, Adrián Neri, J. Porcayo-Calderon

**Affiliations:** 1 CIICAp, Universidad Autonoma del Estado de Morelos, Avenida Universidad 1001, Cuernavaca 62209, Morelos, Mexico; 2 Corrosion y Proteccion (CyP), Buffon 46, Mexico City 11590, Mexico; 3 Department of Chemical Engineering and Metallurgy, University of Sonora, Hermosillo 83000, Sonora, Mexico

**Keywords:** API X-52, sweet corrosion, dialkyl-diamide, coffee bagasse oil, electrochemical techniques

## Abstract

This work reports the performance of a green corrosion inhibitor with double hydrocarbon chain. The evaluated inhibitor was a dialkyl-diamide from coffee bagasse oil and its electrochemical behavior was evaluated on an API-X52 steel in CO_2_-saturated brine at 60 °C. The electrochemical behavior was determined by measurements of open circuit potential, polarization resistance, and electrochemical impedance spectroscopy. In addition, the thermodynamic parameters of the corrosion process were obtained in the temperature range from 40 °C to 80 °C. Electrochemical studies showed that the inhibitor is capable of suppressing metal dissolution by up to 99% at 25 ppm. On the other hand, the thermodynamic parameters indicate that when adding the inhibitor, there is a strong increase in both Ea and ΔH° values, and that as time increases, they decrease until reaching similar values to those observed in the absence of the inhibitor. Furthermore, ΔS° values tend to become more negative with immersion time because of the formation of a stable film on the metal surface.

## 1. Introduction

Amide- and imidazoline-type compounds of fatty acids of vegetable origin (ampholytic compounds) have been found to be excellent corrosion inhibitors because in their structure they have nitrogenous groups and aliphatic chains that act as a metallophilic and hydrophobic group, respectively [1,2,3,4,5,6,7,8]. It has been recognized that the most efficient inhibitors are those with aliphatic chains with 16–18 carbon atoms [9], and since in vegetable oils the largest proportion of their fatty acids correspond precisely to those with 18C chains, it is for this reason that they are used for the synthesis of corrosion inhibitors and the high inhibition efficiencies obtained.

In the hydrocarbon production and transportation industry, one of the main causes of material degradation has been attributed to the presence of dissolved CO_2_ [10,11,12], and its inhibition process has generally been addressed by injecting organic compounds into the corrosive fluid. These molecules are made up of a hydrophilic group (polar group) and a hydrophobic group (hydrocarbon chain). The adsorption of these molecules to the metal surface generates a protective layer by reducing its wettability caused by the hydrophobic group [13,14].

The use of oils obtained from agro-industrial waste has proven to be a sustainable alternative for the development and synthesis of green corrosion inhibitors, and its excellent performance as sweet corrosion inhibitors has been demonstrated [6,15,16,17,18]. Its oils have a content of fatty acids such as stearic, oleic, linoleic, and palmitic, suitable for the synthesis of amide- and imidazoline-type inhibitors, and in particular those inhibitors obtained from coffee bagasse oil have proven to be efficient corrosion inhibitors [4,5,6,7].

On the other hand, it has been shown that the presence of more than one hydrophobic group attached to the polar group can increase the inhibition efficiency [13,19]. Therefore, the idea of this work is to determine the effect of the presence of two hydrocarbon chains on the performance of an inhibitor obtained from coffee bagasse oil as an alternative green inhibitor for sweet corrosion. Its performance has been evaluated on an API-X52 steel in brine (3% by weight NaCl) saturated in CO_2_ at 60 °C by means of open circuit potential, polarization resistance, and electrochemical impedance measurements. In addition, by means of potentiodynamic polarization measurements, the thermodynamic parameters of the corrosion process have been determined in a temperature range from 40 to 80 °C.

## 2. Results and Discussion

### 2.1. Open Circuit Potential Measurements

**Figure 1:**
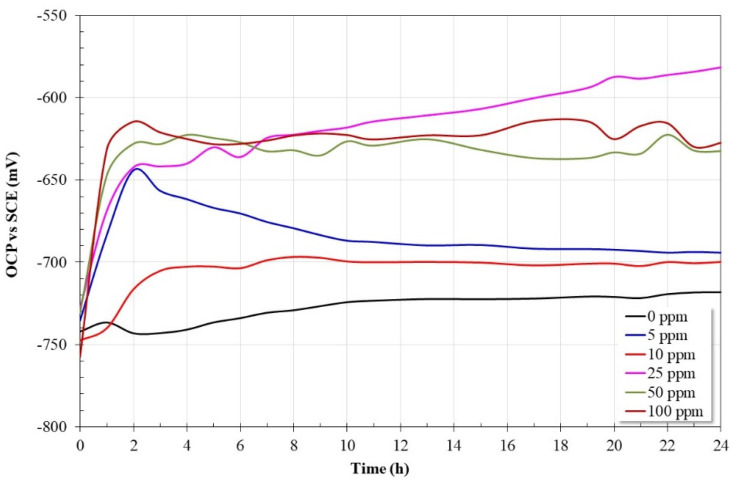
OCP variation for API-X52 steel in CO_2_-saturated brine at 60 °C, with and without inhibitor addition.

Figure 1 shows the variation in OCP values as a function of time for API X52 steel in a 3% NaCl solution saturated with CO_2_ at 60 °C with and without the addition of an inhibitor. In this type of study, the observed trend is indicative of the thermodynamic stability of the metal surface in the electrolyte where it is immersed [20]. It is observed that, in the absence of the inhibitor (0 ppm), the OCP values tend to slowly increase as a function of time from −740 mV to −725 mV throughout the assay. The observed trend indicates that a layer of corrosion products with protective characteristics is possibly being formed on the steel surface since their OCP values change in the noble direction. On the other hand, in the presence of the inhibitor, a rapid increase in OCP values is observed in all cases; this trend suggests that in the presence of the inhibitor, the steel surface showed a more noble behavior due to the adsorption of inhibitor molecules. In general, the trend of OCP values is a function of the inhibitor concentration. With the lowest inhibitor concentration (5 ppm), the OCP values tend to decrease after 2 h, this could be because this concentration is insufficient to form a protective film on the steel surface. At 10, 50, and 100 ppm, the OCP values are practically stable after 2 h of immersion, and with 25 ppm the noblest values were obtained with a tendency to increase as a function of time.

### 2.2. Linear Polarization Resistance Measurements

**Figure 2:**
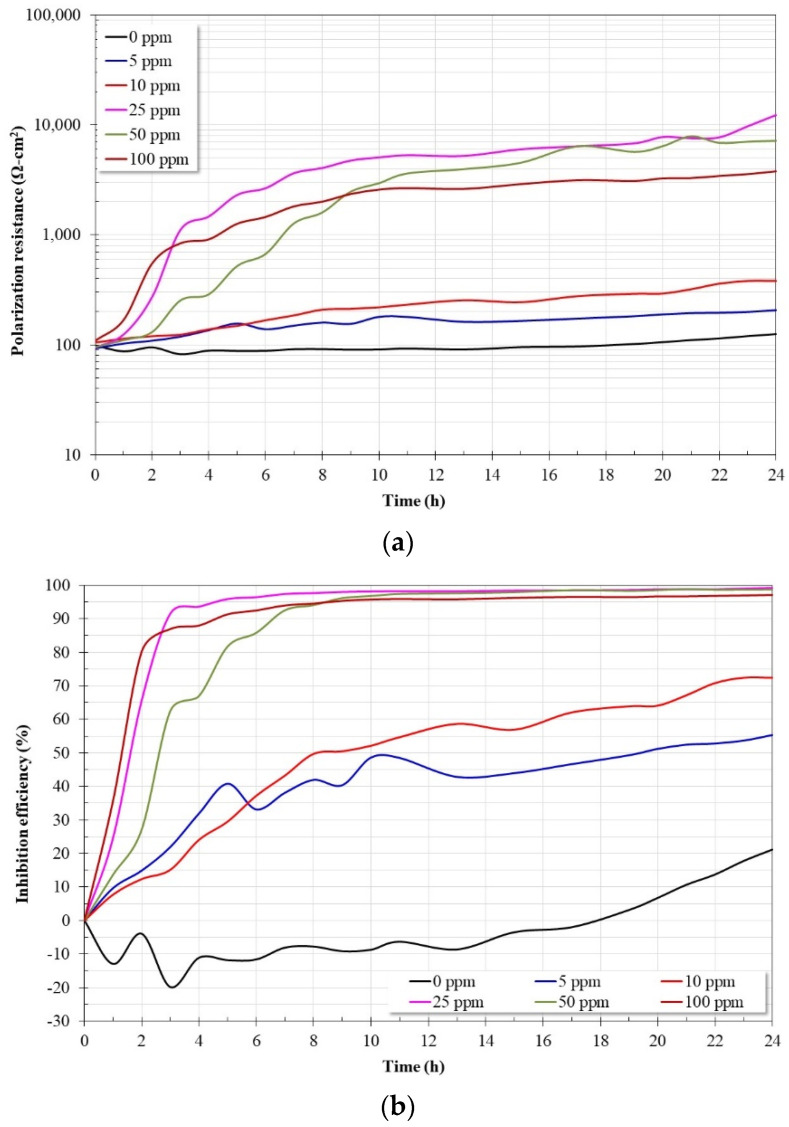
(**a**) Rp variation for API-X52 steel in CO_2_-saturated brine at 60 °C, with and without inhibitor addition. (**b**) Inhibition efficiency as a function of inhibitor concentration.

Figure 2a shows the variation in the Rp values as a function of time for the API X52 steel in a 3% NaCl solution saturated with CO_2_ at 60 °C with and without the addition of the inhibitor. Since the value of the resistance to polarization is inversely proportional to the corrosion rate, it is common to interpret that the trend of these values indicates whether the material is undergoing an active corrosion process (continuous decrease in the Rp value), the material remains immune, or a stable protective film is present (constant Rp values), or a protective film is developing on the surface (continuous increase in Rp values).

Based on the above, it can be established that, in the absence of the inhibitor, the Rp values remain almost constant in the first 6 h of immersion, but subsequently they increase slightly until the end of the test. This may be indicative of the formation of stable and protective corrosion products based mainly on iron carbonates. This is consistent with what has been reported in the literature, where it has been indicated that from 60 °C, iron carbonate with protective properties begins to precipitate on the steel surface [21]. In the presence of the inhibitor, the Rp values indicate that a protective film is developing on the metal surface due to the adsorption of the inhibitor molecules, forming a barrier that limits the entry of aggressive species. The protective capacity of the inhibitor increases when its concentration increases from 5 ppm to 25 ppm, and above this concentration (50 and 100 ppm) the resistance values decrease. The decrease in Rp values is possibly due to its concentration being too high. This causes repulsive forces that limit the free access of the molecules to the metal surface or cause the detachment of already adsorbed molecules. At this temperature, the optimal dose of the inhibitor is 25 ppm. The Rp values obtained are considerably larger than those reported for similar inhibitors [17,19].

In Figure 2b, the inhibition efficiency derived from the data in Figure 2a is shown. The inhibition efficiency values were determined according to the following relationship:

(1) $$E\left(\%\right)=\frac{{\mathrm{Rp}}_{i}-{\mathrm{Rp}}_{b}}{{\mathrm{Rp}}_{i}}\times 100$$

where R_pb_ is the value of Rp in the absence of the inhibitor and R_pi_ is the value of Rp in the presence of the inhibitor. From the figure in the absence of the inhibitor after an initial corrosion process, the formation or precipitation of corrosion products on the surface of the steel caused a decrease in its corrosion rate, observing a reduction close to 20% at the end of the test. However, with the addition of 5 and 10 ppm of the inhibitor, an inhibition efficiency of the order of 50–70% was obtained, but at higher concentrations the efficiency increased above 95%. The trend indicates that the maximum protection efficiency is reached with 25 ppm of the inhibitor with an inhibition efficiency of 99%. At this concentration, the greatest surface coverage is achieved, which limits the free contact of the electrolyte with the metal surface and makes diffusion through the protective layer the limiting step in the metal dissolution process [22].

The inhibition efficiencies are higher than those obtained with similar inhibitors obtained from other vegetable oils [17,19] and imidazolines with more than one hydrocarbon chain [13]. The higher inhibition efficiencies may be due to the presence of unsaturations in the hydrocarbon chains that favor the adsorption of the inhibitor to the metal surface [14,23,24].

**Figure 3:**
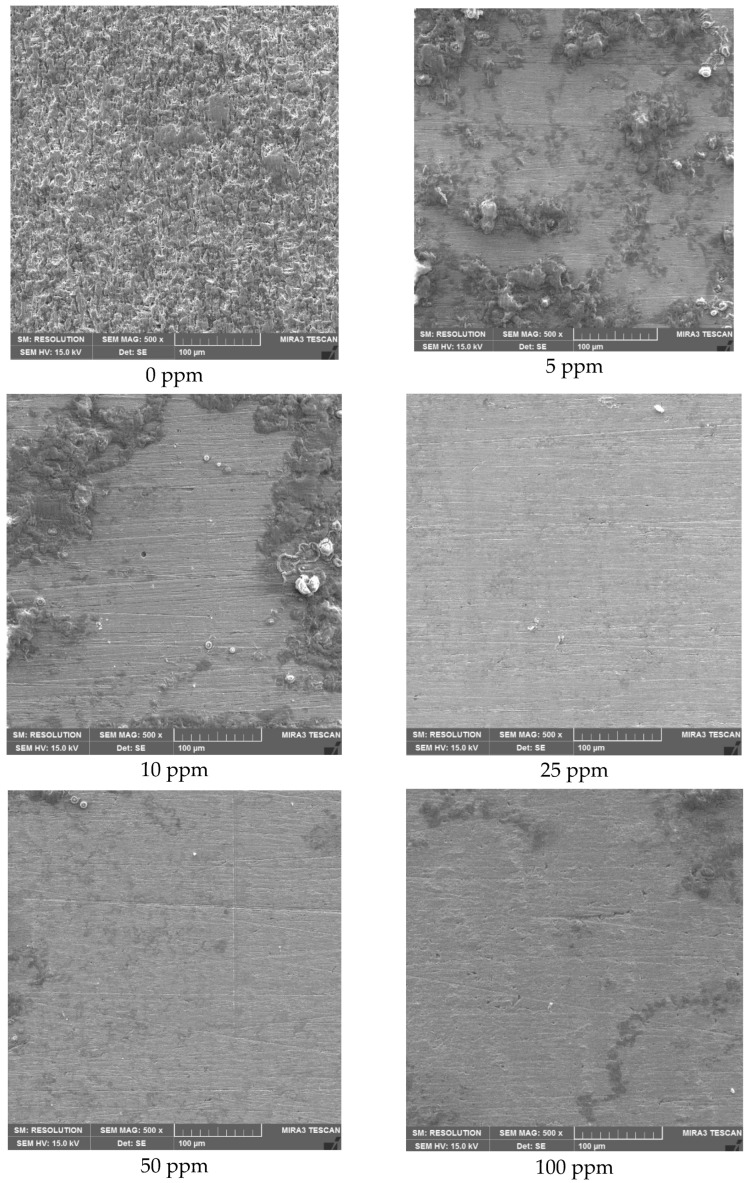
Morphological aspects of the API-X52 steel surface after the corrosion test in CO_2_-saturated brine at 60 °C.

Figure 3 shows the morphological aspects of the clean surface of the evaluated steel in the presence of the different inhibitor concentrations. It is evident that the greatest damage observed corresponds to the condition in the absence of the inhibitor. At low concentrations of the inhibitor (5 and 10 ppm), the surface showed strongly adhered corrosion product residues, and at high concentrations (50 and 100 ppm) the concentration of residual corrosion products was lower. It is clear that at 25 ppm the surface of the steel is practically free of surface defects due to the corrosive action of the electrolyte.

### 2.3. Electrochemical Impedance Spectroscopy

**Figure 4:**
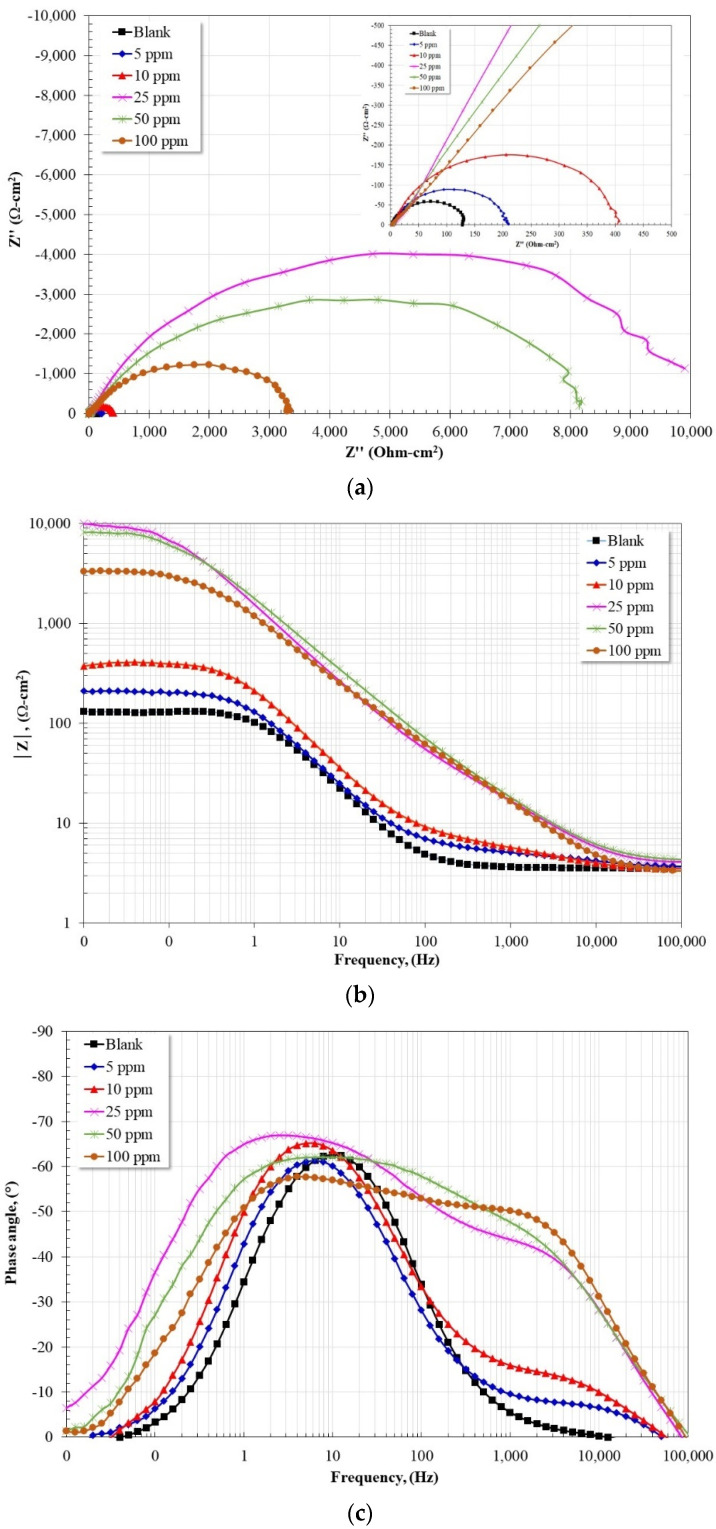
Nyquist (**a**) and Bode (**b**,**c**) plots for API-X52 steel in CO_2_-saturated brine after 24 h immersion at 60 °C, with and without inhibitor addition.

Figure 4 shows the Nyquist and Bode diagrams for API X52 steel in a 3% NaCl solution saturated with CO_2_ at 60 °C with and without the addition of the inhibitor after 24 h of immersion. According to the Nyquist diagram, in all cases, the apparent presence of a single capacitive semicircle is observed, the diameter of which increases as the inhibitor dose increases up to 25 ppm, and at higher concentrations it tends to decrease. Since the diameter of a capacitive semicircle is equal to Rct + Rs, the observed trend is consistent with the Rp values observed at 24 h (Figure 2a). According to the Bode plot in its impedance modulus format, in the absence of the inhibitor the presence of both high and low frequency plateaus is observed, and a linear relationship in the intermediate frequency region. However, in the presence of the inhibitor, the high-frequency plateau is formed at frequencies greater than 10 kHz, and at lower frequencies the presence of two linear relationships (log |*Z*| − log *f*) is observed. In the low frequency region, the low frequency plateau is observed, whose value increases with increasing inhibitor concentration up to 25 ppm and at higher concentrations it tends to decrease. The trend observed in the maximum value of the low-frequency plateau coincides with the trend of the Rp values (Figure 2a). On the other hand, according to the Bode diagram in its phase angle format, in the absence of an inhibitor, the formation of a time constant is detected, whose maximum is located around 10 Hz with a value of 63°. In the presence of an inhibitor, two-time constants are observed, where the first is in the high frequency region with a maximum phase angle between 45–50° for concentrations greater than 10 ppm, and between 1–10 Hz the presence of the second time constant is noted, with a maximum phase angle around 67° with the addition of 25 ppm of the inhibitor. Due to the location of the first time constant (high frequency region) it can be associated with the formation of a thin layer of the inhibitor molecules onto the steel surface, and because in the low frequency region the angle of phase tends to 0°, it can be suggested that the inhibitor film is dense, which significantly reduced the diffusion of aggressive species of the electrolyte towards the metal surface.

**Figure 5:**
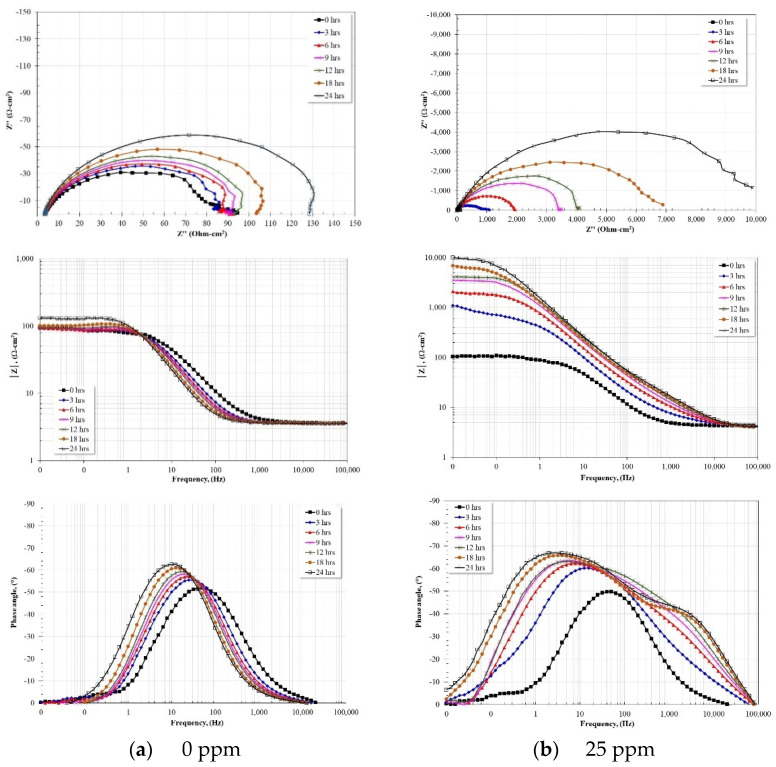
(**a**) Nyquist and Bode plots for API-X52 steel in CO_2_-saturated brine a 60 °C. (**b**) Nyquist and Bode plots for API-X52 steel in CO_2_-saturated brine a 60 °C and 25 ppm of inhibitor.

Figure 5 shows the evolution of the Nyquist and Bode diagrams for the steel in the absence of the inhibitor (Figure 5a) and with 25 ppm of the inhibitor (Figure 5b), which, according to the results, showed the best inhibition efficiency.

In the absence of the inhibitor (Figure 5a), the Nyquist diagram shows the evolution of a capacitive semicircle whose diameter apparently increases with immersion time. The increase in diameter may be associated with the formation and/or precipitation of corrosion products on the steel surface, which reduced the reaction area. The Bode plot in its impedance modulus format shows the typical behavior of a corrosion process with a single time constant, that is, formation of the high-frequency plateau starting at 1 kHz, a linear relationship (log |*Z*| − log *f*) in the intermediate frequency region, and the presence of the low-frequency plateau. According to the value of the impedance module, |*Z*|, the increase in the value of Rct is negligible as the immersion time elapses. This suggests a low protective capacity of the corrosion products formed on the steel surface. The Bode plot in its phase angle format shows only one time constant, which agrees with the formation of a single capacitive semicircle and a single log |*Z*| − log *f* linear relationship in the intermediate frequency region. The maximum of the phase angle tends to increase (52 → 63°) and move to lower frequencies (50 → 10 Hz) with the immersion time. This behavior can be associated with the formation of a layer of corrosion products on the steel surface.

On the other hand, with the addition of 25 ppm of the inhibitor (Figure 5b), the Nyquist diagram shows the apparent presence of a single capacitive semicircle whose diameter increases significantly with immersion time. The magnitude of the increase in the diameter of the capacitive semicircle is associated with the adsorption of the inhibitor on the steel surface, thereby forming a protective layer that isolates the metal surface from the aggressive electrolyte. From the Bode diagram in its impedance modulus format, after the addition of the inhibitor, the high-frequency plateau begins to form at frequencies greater than 10 kHz and in that region a linear relationship, log |*Z*| − log *f*, is formed. The appearance of this linear relationship in this range of frequencies is a characteristic sign of the adsorption of organic inhibitors with a hydrophobic group formed by a hydrocarbon chain in the structure of their molecule [4,6,15,23]. In the intermediate frequency region, the presence of another linear relationship is observed, log |*Z*| − log *f*, whose extension increases with immersion time from 10 Hz to approximately 0.1 Hz. In the low-frequency region, the low-frequency plateau is observed, whose magnitude of the impedance modulus increases with immersion time up to approximately two orders of magnitude at the end of the test. This increase is due to the increase in resistance to load transfer due to the adsorption and formation of a protective film on the steel surface. From the Bode diagram in its phase angle format, in the high frequency region the formation and evolution of the first-time constant associated with the protective layer of the inhibitor adsorbed on the steel surface is observed. Its location is around 2 kHz, reaching a maximum phase angle of 42° after approximately 24 h of immersion. The presence of this time constant is also a characteristic of organic inhibitors with a hydrophobic group formed by a hydrocarbon chain [4,6,15,23], however, its position and magnitude of maximum phase angle is lower than reported. In the intermediate frequency region, the presence and evolution of the second time constant is observed, which corresponds to the capacitive response of the metallic surface. It is observed that this time constant moves from 40 Hz to 2 Hz, and its maximum phase angle increases from 50° to 68° at the end of the test. This behavior is characteristic of organic inhibitors where much of the molecule adsorbs in a planar fashion [14,25]. The characteristics of the two-time constants suggest that a large part of the inhibitor molecule was adsorbed in a planar manner and only part of the length of the hydrocarbon chains was directed towards the electrolyte.

**Figure 6:**
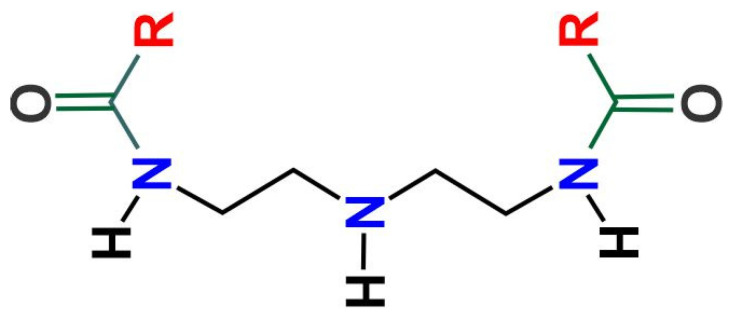
Chemical structure of the dialkyl-diamide of coffee bagasse oil (R = alkyl chains).

Taking into account the structure of the inhibitor (Figure 6), it is possible that its adsorption to the metal surface was carried out from the N-rich group (diaminodiethylamine group) to the unsaturations present in the hydrocarbon chains [1,14,23,24].

**Figure 7:**
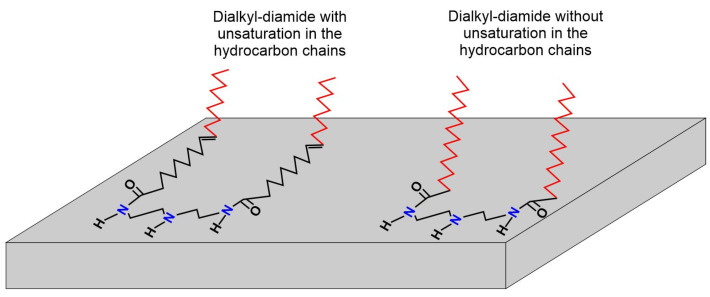
Schematic of the inhibitor adsorption mode onto metal surface.

It has been reported that the inhibitors with two hydrophobic chains show higher inhibition efficiencies than those observed with the inhibitors of one and three hydrophobic chains [13]. In addition, the high content of hydrocarbon chains with unsaturations (≈57%) and short hydrocarbon chains (≈36%) could favor the phase angles observed in both time constants, and their adsorption mode could occur as suggested in Figure 7.

The proposed scheme is consistent with the findings reported by M.A.J. Mazumder et al. [13] and J. Porcayo-Calderon et al. [14]. On the one hand, despite the fact that the presence of NaCl and CO_2_ affects the optimal concentration of inhibition, the inhibition mechanism is based on the displacement of the water molecules adsorbed to the metal surface due to the coordinate bond existing between the empty sites of the d-orbitals of Fe, and the π-electrons from the N centers, and on the other hand, the surface coverage is increased by the presence of the existing double bonds in the hydrocarbon chains.

**Figure 8:**
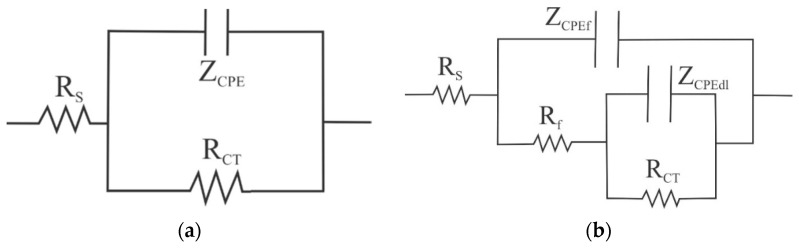
Equivalent circuits: (**a**) without inhibitor; (**b**) with inhibitor.

Based on the behavior observed in the evolution of the impedance spectra, Figure 8 shows the equivalent circuits proposed to model the electrochemical behavior of the X52 steel, both in the absence and in the presence of the corrosion inhibitor. In this case, Rs represents the electrolyte resistance, Rct the charge transfer resistance, Rf the resistance of the adsorbed inhibitor film, Z_CPEdl_ the impedance of the constant phase element (CPE) of the double layer, and Z_CPEf_ the impedance of the constant phase element of the adsorbed inhibitor film.

In general, it is common to use the CPE instead of a capacitor to compensate for surface irregularities and non-uniform distribution of the charge transfer. Generally, in these cases, the capacitive semicircles appear depressed, and the degree of depression depends on the phase of the CPE [26]. The impedance of the CPE is dependent on the frequency and is defined by the expression:

(2) $$Z_{\mathrm{CPE}}=\frac{1}{Y_{0}{\left(i\omega \right)}^{n}},$$

**Figure 9:**
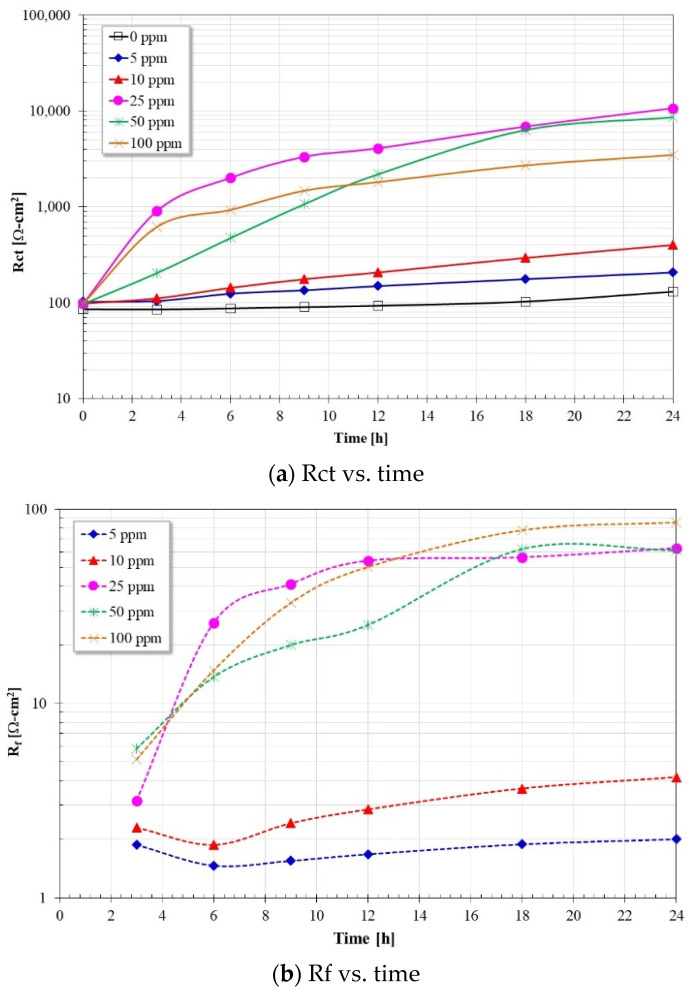
Rct and Rf values as a function of time and inhibitor concentration.

**Figure 10:**
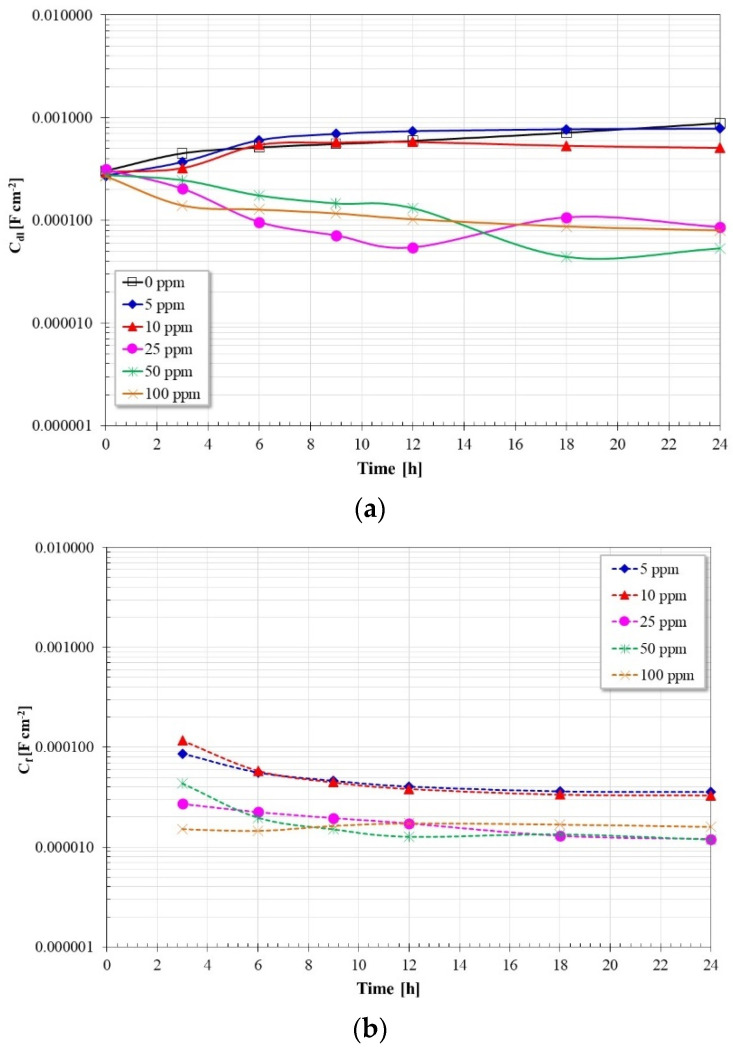
Cdl and Cf values as a function of time and inhibitor concentration.

In this case, Yo = proportionality factor; i = √−1, ω = 2π*f* = angular frequency, *f* = frequency, and *n* = slope of the relation log |*Z*| − log *f*. If *n* = 1, CPE represents an ideal capacitor where Yo = capacitance, and if *n* = 0.5, CPE represents a Warburg impedance with diffusional character. The values of the capacitance of the double layer and of the inhibitor film can be obtained from the value of Yo according to [27]:

(3) Ci=(Y0iRi(1−ni))1ni,

Based on the proposed models, the fit of the impedance spectra showed the results of Figure 9 and Figure 10. Figure 9a shows a high coincidence of the calculated Rct values with those Rp values obtained from the measurements of resistance to linear polarization (Figure 2a). This suggests that the proposed equivalent circuits correctly represent the electrode processes occurring on the steel surface. The Rct values are higher than those reported with similar inhibitors obtained from other vegetable oils [19] and imidazolines with more than one hydrocarbon chain [13]. On the other hand, from Figure 9b the Rf values are lower than those of Rct, and its value is higher at concentrations greater than 10 ppm, reaching maximum values between 60–80 ohms-cm^2^.

Regarding the capacitance values, according to Figure 10a, in the absence of the inhibitor, the Cdl values show an increase with immersion time, however, for concentrations of 5 and 10 ppm, the Cdl values show approximately the same values as in the absence of the inhibitor. At concentrations greater than 10 ppm, the Cdl values are lower and tend to decrease with immersion time until reaching approximately the same value. The trend of these values is inversely proportional to the values of Rct, that is, as the values of Rct increase, the values of Cdl tend to decrease. Furthermore, according to the Cdl definition:

(4) $$C_{\mathrm{dl}}=\frac{{\varepsilon \varepsilon }_{o}A}{d}$$

If the terms ε_o_ (vacuum dielectric constant) and *A* (reaction area) are constant, the only terms that favor a decrease in capacitance are a decrease in the dielectric constant (ε) of the adsorbed film and/or an increase in the thickness of the double layer (*d*). Therefore, since the inhibitor molecules (low dielectric constant) have replaced the water molecules (high dielectric constant) on the steel surface, this favored the decrease in Cdl values, as well as an increase in the double layer thickness [14,28,29,30]. On the other hand, according to Figure 10b, the C_f_ values are lower than the Cdl and show a tendency to decrease with immersion time. For inhibitor concentrations greater than 10 ppm, their values are the lowest and very close to each other, according to the definition of capacitance, this suggests that the thickness of the inhibitor film adsorbed to the metal surface is greater for these concentrations.

### 2.4. Potentiodynamic Polarization

Once the optimal inhibition concentration was determined, potentiodynamic polarization curves were performed at different immersion times and temperatures. In all cases, for each polarization curve a new working electrode was used.

**Figure 11:**
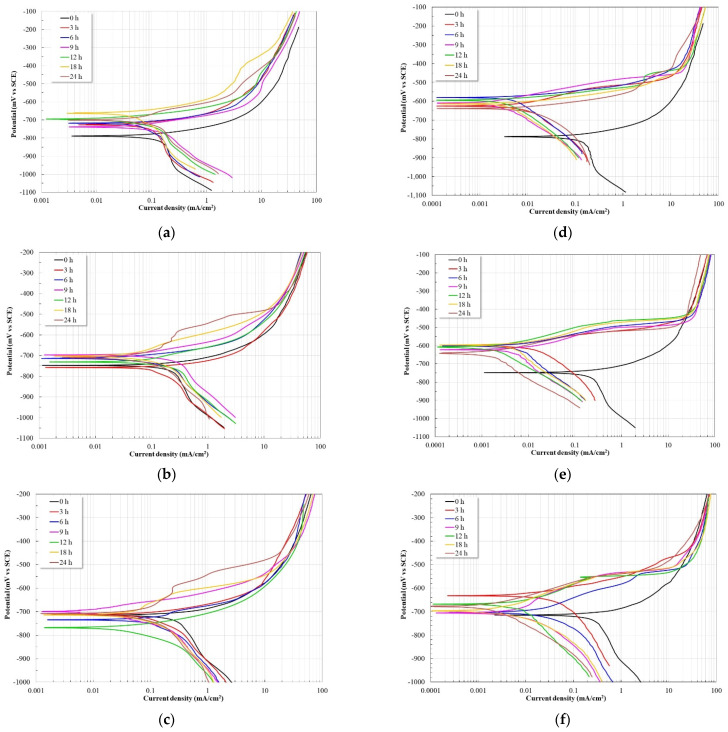
Potentiodynamic polarization curves, as a function of time and temperature, for API-X52 steel in CO_2_-saturated brine, without and with 25 ppm of inhibitor. (**a**) 40 °C, 0 ppm. (**b**) 60 °C, 0 ppm. (**c**) 80 °C, 0 ppm. (**d**) 40 °C, 25 ppm. (**e**) 60 °C, 25 ppm. (**f**) 80 °C, 25 ppm.

Figure 11a shows the polarization curves at 40 °C in the absence of the inhibitor; it is observed that regardless of the immersion time, all the curves show the same behavior. The anodic branch shows that the steel underwent an active corrosion process, that is, large increases in current density by slightly increasing the potential. This behavior is characteristic of materials that are not capable of developing a protective oxide. A displacement of the polarization curves to nobler corrosion potentials is observed as time elapses until 18 h to subsequently decrease. This can be associated with the inability of the material to develop a stable protective oxide. On the other hand, the corrosion current density values only show slight oscillations and are within the same order of magnitude. This confirms that at 40 °C the steel undergoes an active corrosion process and is incapable of developing a stable passive layer. At 60 °C (Figure 11b), the polarization curves also show a similar behavior regardless of the immersion time. However, after 3 h of immersion, a shift in the corrosion potential is observed towards nobler values with a slight shift to lower current densities. The anodic branch shows an active corrosion process, although an increase in its slope is observed with increasing immersion time. This may be associated with the precipitation of protective layers on the surface of the working electrode. It is known that from 60 °C the formation of protective layers based on iron carbonate become more adherent and protective, thereby reducing the corrosion rate [31]. At 80 °C (Figure 11c), the polarization curves show a slight shift to lower current densities but with oscillations in its corrosion potential, being more active at 3 and 12 h. Until 12 h, the anodic branch shows an active corrosion process and after 18 h an increase in the slope of the anodic branch is observed, which may correspond to the formation of protective corrosion products on the surface of the working electrode.

However, in the presence of the inhibitor at 40 °C (Figure 11d), it is observed that after 3 h of immersion, the polarization curves have shifted to more noble potentials (around 250 to 300 mV with respect to Ecorr) and at lower current densities (about an order of magnitude lower). These changes show that the inhibitor has adsorbed on the surface of the working electrode acting as a barrier between the electrolyte and the metal surface, since both the anodic and cathodic currents have decreased. However, even though the anodic branch shows an increase in its slope, it is observed that with an increase of around 100 mV with respect to the corrosion potential, large increases in current density are generated. This is possibly due to detachment of the inhibitor due to low adsorption forces. On the other hand, at 60 °C (Figure 11e), once again it is observed that after 3 h of immersion, the polarization curves show a shift to more noble potentials (around 100 to 150 mV with respect to Ecorr), and at lower current densities (about two orders of magnitude). These displacements indicate inhibitor adsorption and increased protection of the working electrode. The anodic branch shows a greater increase in its slope than that observed at 40 °C, however around 150 mV above Ecorr a large increase in current density is observed, indicating desorption of the inhibitor film. At 80 °C (Figure 11f), it is observed that after 3 h of immersion, a displacement of the polarization curves occurs to nobler potentials and lower current densities (at least by an order of magnitude). However, it is observed that the corrosion potentials keep oscillating, possibly due to adsorption–desorption processes of the inhibitor molecules. It is possible that at this temperature the concentration of the inhibitor is not sufficient to guarantee the protection of the working electrode. As at 60 °C, the anodic branch shows an increase in its slope, being more noticeable after 9 h of immersion. Similarly to what was observed at 60 °C, around 150 mV above Ecorr there is an increase in current density, which may correspond to the desorption of the inhibitor film.

In general, with the addition of the inhibitor, a shift to lower current densities was observed, which in turn caused a change in both slopes (anodic and cathodic), indicating a decrease in both the anodic reaction rate (metallic dissolution) and the cathodic reaction rate (H_2_ evolution) [32,33].

**Figure 12:**
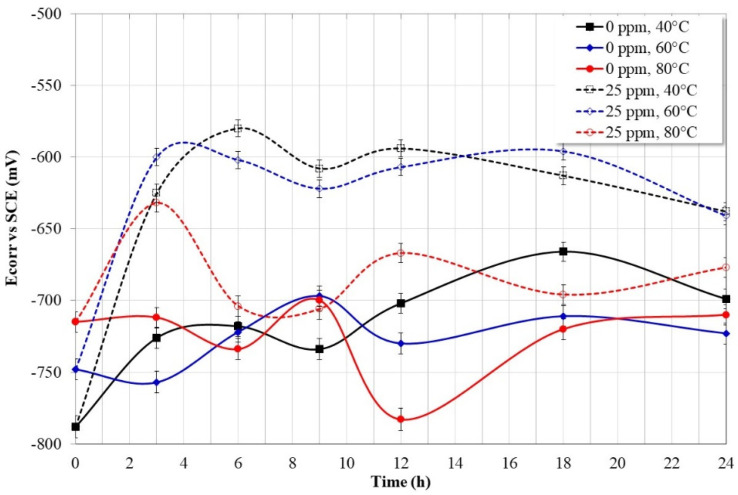
Corrosion potential variation for API-X52 steel in 3% NaCl solution saturated with CO_2_, determined from potentiodynamic polarization curves.

Figure 12 shows the variation in the Ecorr values obtained from the potentiodynamic polarization curves. In the absence of the inhibitor, it is observed that the Ecorr values are more active with increasing temperature, this indicates that the material becomes more susceptible to corrosion. However, in the presence of the inhibitor, Ecorr values tend to be more noble, indicating a tendency to protect. This is more evident at 40 °C and 60 °C, however at 80 °C oscillations in the Ecorr values are observed, possibly due to adsorption–desorption processes of the inhibitor molecules.

**Figure 13:**
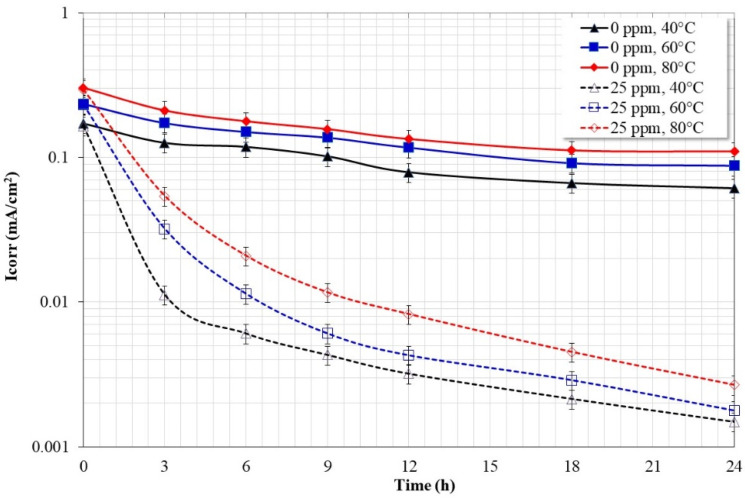
Corrosion current density variation for API-X52 steel in 3% NaCl solution saturated with CO_2_, determined from potentiodynamic polarization curves.

Figure 13 shows the variation in the Icorr values, obtained from the potentiodynamic polarization curves, as a function of time and temperature. From the graph in the absence of the inhibitor the steel shows Icorr values of the same order of magnitude regardless of the test temperature, with a tendency to decrease with immersion time and increase with temperature. On the other hand, in the presence of the inhibitor, it is observed that the Icorr values tend to decrease by more than one order of magnitude in the first 3 h of immersion and subsequently remain in the same order of magnitude regardless of the temperature. Since the values of Icorr are inversely proportional to the resistance to polarization (Rp), it is observed that the trend in the values of Icorr adjusts to that observed with the values of Rp (Figure 2a).

**Figure 14:**
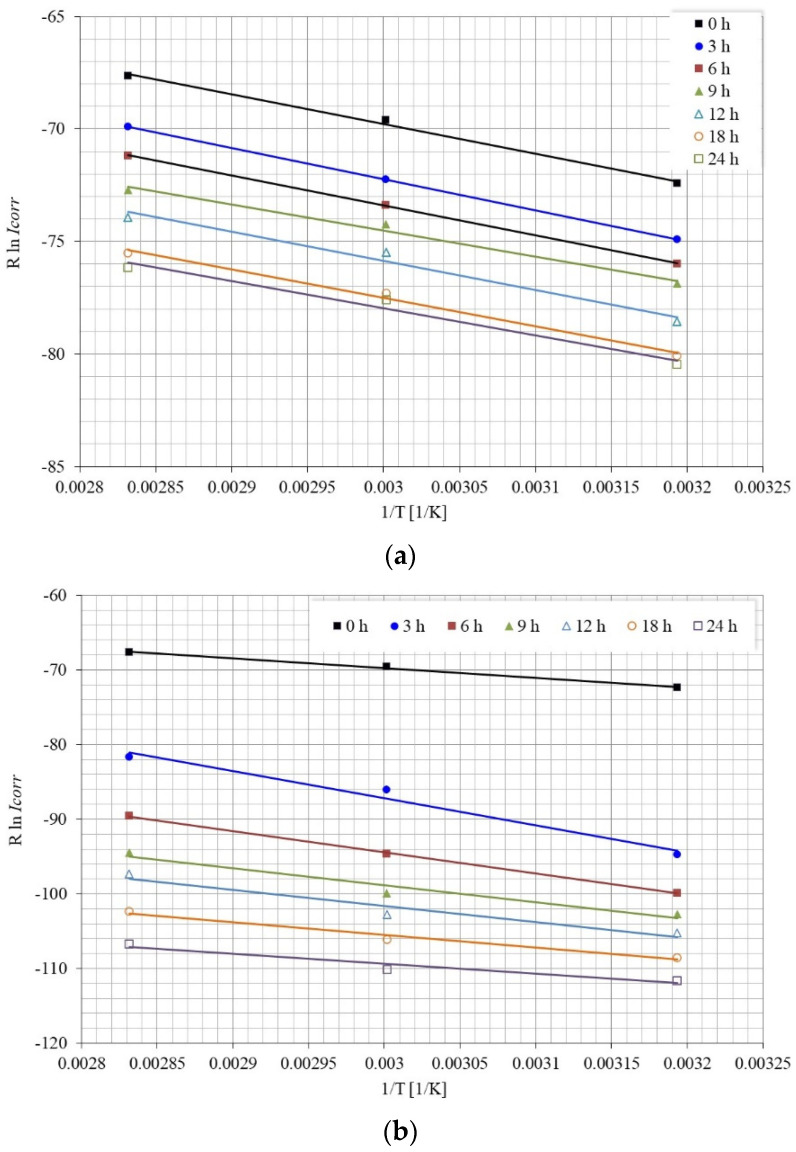
Arrhenius plot, for the calculation of Ea, as a function of immersion time: (**a**) without inhibitor, (**b**) with 25 ppm inhibitor.

The effect of temperature on the corrosion process can be explained with the use of the Arrhenius equation, where the activation parameters of both the anodic branch and the cathodic branch can be calculated by means of the following equation [34,35]:

(5) $$\mathrm{Icorr}=k\exp\left(-\frac{E_{a}}{\mathrm{RT}}\right)$$

where Ea (J mol^−1^) is the activation energy of the corrosion process; R the universal gas constant (J mol^−1^ K^−1^); k a constant; and Icorr is the corrosion current density (A cm^−2^). The activation energy in the absence and presence of the inhibitor was determined by the slope of the graph of Rln(Icorr) against 1/T (Figure 14), and the results obtained are shown in Table 1.

**Figure 15:**
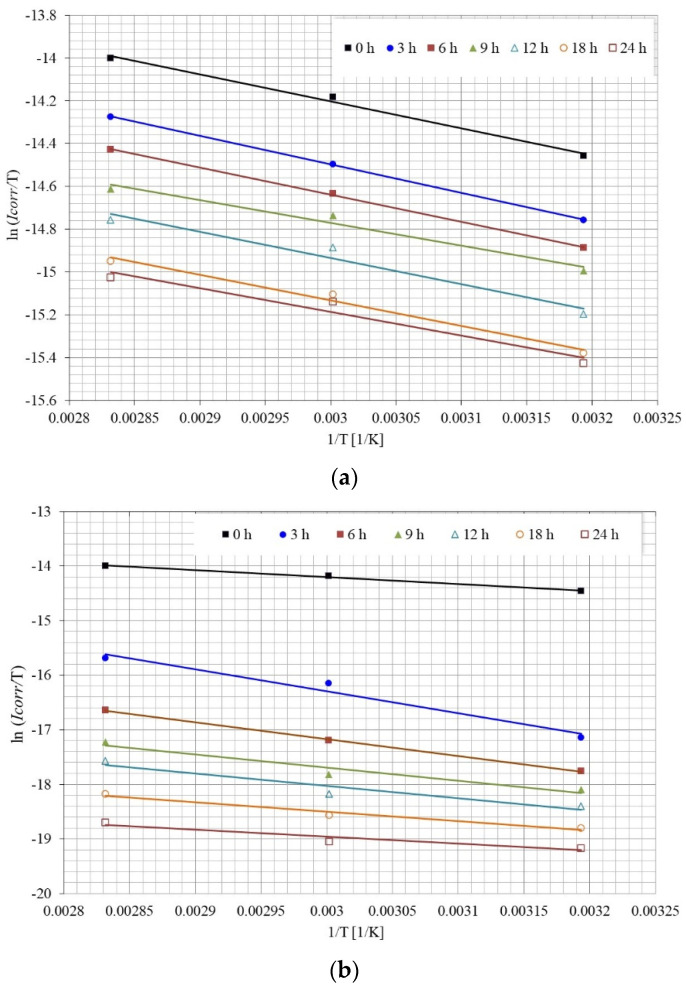
Arrhenius plot, for the calculation of ΔH° and ΔS°, as a function of immersion time: (**a**) without inhibitor, (**b**) with 25 ppm inhibitor.

The enthalpy (ΔHa°) and entropy (ΔSa°) change can be obtained by the transition state equation, an alternative form of the Arrhenius equation [22,34,36]:

(6) $$I=\frac{\mathrm{RT}}{\mathrm{Nh}}\exp\left(\frac{\Delta S{}^{\circ}}{R}\right)\exp\left(-\frac{\Delta H{}^{\circ}}{\mathrm{RT}}\right)$$

where h is Planck’s constant (6.626176 × 10^−34^ J s); N Avogadro’s number (6.023 × 10^23^ mol^−1^). Plotting ln(Icorr/T) against 1/T yields a straight line whose slope is, (−∆H°/R), with intercept of (ln(R/Nh) + ΔS°/R) (Figure 15). Table 1 reports the values of Ea, ΔH°, and ΔS°, as a function of the immersion time of the corrosion process without the addition of the inhibitor and with the addition of 25 ppm of the inhibitor.

According to the calculated values, it is observed that in the absence of the inhibitor, the activation energy oscillated between 12 and 14 kJ/mol during the entire test. The magnitude of Ea represents the energy barrier necessary for metal dissolution to take place. Ea values around 60–80 kJ/mol have been reported for the dissolution of Fe in H_2_SO_4_ solutions [22,33,36,37], and about 40 kJ/mol in HCl solutions [38]. In electrolytes such as those evaluated here, a wide range of Ea values is reported, ranging from 8 to 23 kJ/mol [39,40,41,42,43,44,45] up to 30–33 kJ/mol [2,3,24], depending on the NaCl content of the brine, in general being that the lowest values are reported for concentrations below 3.5% and the highest for concentrations of 5%. The low activation energy determined here suggests a greater aggressiveness of the CO_2_-saturated brine electrolyte, possibly due to a greater conductivity due to the ionic species present.

However, in the presence of the inhibitor, the activation energy increased to 36.3 kJ/mol two hours after the inhibitor had been added, and subsequently its value tended to decrease until it reached a value similar to that obtained in the absence of the inhibitor. The increase in the value of Ea has been associated with an initial stage of physical adsorption of the inhibitor, and its decrease or low variation to a chemisorption process, considering that part of the energy is used for the chemical reaction [22,33,37]. Other studies suggest that the decrease in the value of Ea is due to a slow adsorption of the inhibitor or to a change in the net corrosion reaction between the unprotected part with respect to the protected one, that is, the reaction speed in the protected area is substantially less than in the unprotected area [22,38].

However, it should be noted that many of the studies report the calculation of Ea from measurements made after adding the inhibitor to the electrolyte and its magnitude with respect to the value of Ea without the inhibitor is associated with the type of adsorption of the inhibitor (physical or chemical). For example, for the dissolution of Fe in acid media, increases in the value of Ea are reported and it is associated with an increase in the energy barrier for corrosion to take place [33,36,37,38], and others report a decrease and associate it with a chemisorption process [22]. In media similar to the one used here, increases (30–50 kJ/mol) have been reported, and these have been associated with a physisorption process, an increase in the energy barrier, or an increase in the thickness of the double layer [24,39,40,43,45], and decreases (5–20 kJ/mol), to a chemisorption process [2,3,24,41,42,44].

It has also been observed [24] that the Ea value initially increases and subsequently decreases with the increase in inhibitor concentration, and this has been associated with a change in the adsorption mode, from physical to chemical adsorption. At low concentrations, there is a blockage by physisorption of the active sites because the three N atoms (positive charge) interact with the metallic surface (negative charge), in addition to the fact that the double bonds of the hydrocarbon chain can also interact with the surface through its π electrons, and with an increasing concentration the electron-rich amine groups block the active sites by chemisorption.

Then, based on the above, it can be said that the observed increase in Ea values is due to the fact that the inhibitor is initially adsorbed by a physical adsorption process and the subsequent decrease is due to a change in the type of adsorption, that is say from physical adsorption to chemical adsorption.

Regarding the activation enthalpy, in the absence of the inhibitor, values between 9–11 kJ/mol were obtained during the test period. The positive values obtained indicate that the metal dissolution process is endothermic [22], ΔH° values around 60 kJ/mol have been reported for the dissolution of Fe in H_2_SO_4_ solutions [22,36], and 40 kJ/mol in HCl solutions [38]. In media similar to those evaluated here, a wide range of ΔH° values is reported, ranging between 5–15 kJ/mol [42,43,44]. The low enthalpy value determined here also suggests that the dissolution of Fe in CO_2_-saturated brine is an endothermic process.

On the other hand, in the presence of the inhibitor, ΔH° increases up to 33.5 kJ/mol after two hours of the inhibitor having been added and subsequently its value tends to decrease until it reaches a value similar to that obtained in the absence of the inhibitor. ΔH° values for the dissolution of Fe in acidic media are reported shortly after adding the inhibitor in the order of 50–90 kJ/mol [22,36,38]. In corrosive media similar to the one used here, ΔH° values of the order of 5–33 kJ/mol have been reported [42,43,44]. In general, in both cases (with and without inhibitor) it is observed that the Ea and ΔH° values show the same trend as well as very similar values. This agrees with the proposal that for a chemical reaction in solution both values should theoretically be equal [44].

Finally, with respect to entropy, in the absence of the inhibitor, ΔS° values ranging between −280 and −300 J/mol-K were obtained throughout the test. Positive values have been associated with an increase in the order of the activated complex and that this is the rate-limiting step and that it represents an association step [22,34,36,38]. ΔS° values between −50 and −120 J/mol-K have been reported for the dissolution of Fe in H_2_SO_4_ solutions [22,36], and about −180 J/mol-K in HCl solutions [38]. In media similar to the one used here, ΔS° values of the order of −150 to −170 J/mol-K have been reported [42,44].

In the presence of the inhibitor, ΔS° increases to −232 J/mol-K after two hours of being added and subsequently its value tends to decrease constantly up to a value of −323 J/mol-K at the end of the test. ΔS° values for the dissolution of Fe in acid media are reported shortly after adding the inhibitor between −50 and −120 J/mol-K [22,36,38]. In media similar to the one used here, ΔS° values of the order of −160 and −221 J/mol-K have been reported [42,44]. The negative values of ΔS°, with and without the presence of the inhibitor, suggest that the complex activated in the rate-limiting step represents an associative rather than a dissociative step, and this implies an increase in order as the reaction goes from reagents to the activated complex [42,44]. It has also been reported that when the concentration of the inhibitor increases, the ΔS° values decrease due to a higher order of the Fe-inhibitor complex, and that when its value is more negative it is because the metallic surface is more homogeneous. This because of a more orderly and stably adsorbed inhibitor film [44]. Then, based on the above, the trend observed in this study suggests that the inhibitor adsorption increases with immersion time, forming a highly ordered and stable film on the steel surface.

## 3. Materials and Methods

The tests were carried out with cylindrical working electrodes (reaction area 4.53 cm^2^) made of API-X52 steel. Prior to each test, the working electrodes were roughened with emery paper up to 600 grits, and later washed with distilled water, and finally cleaned with acetone and dried.

For the electrochemical tests, an electrochemical cell with three electrodes was used, where the reference electrode was one of saturated calomel (SCE), a graphite rod was used as the auxiliary electrode, and API-X52 steel was used as the working electrode (WE). The corrosion tests were carried out at 60 °C in a sodium chloride solution (3% by weight) saturated with CO_2_.

The evaluated inhibitor was a dialkyl-diamide from coffee bagasse oil that, according to its synthesis process [17,19,46,47,48], has a general structure as shown in Figure 6. The diaminodiethylamine group of the molecule is the electron-rich hydrophilic section that promotes its adsorption to the metal surface and hydrocarbon chains is the hydrophobic region that decreases the wettability of the steel surface [13].

The inhibitor is a mixture of dialkyl-diamide molecules where 49% corresponds to linoleic acid, 36% to palmitic acid, 8% to oleic acid, and 7% to stearic acid. The proportion of hydrocarbon chains is consistent with the composition of fatty acids reported for similar inhibitors in previous studies [4,5,6,7]. The concentration of inhibitor used in the corrosion tests was 5, 10, 25, 50, and 100 ppm.

Inhibitor performance was determined by open circuit potential (OCP), linear polarization resistance (LPR), and electrochemical impedance spectroscopy (EIS) measurements. The OCP measurements were made at one-hour intervals for 24 h, the LPR measurements, at one-hour intervals for 24 h, for which the working electrode was polarized ±10 mV with respect to its open circuit potential at a scan rate of 10 mV/min, and EIS studies applying a 10 mV peak-to-peak signal with respect to the OCP value in the frequency range of 100 kHz to 0.01 Hz.

Finally, because it has been observed that the amide and imidazoline inhibitors derived from fatty acids reach their maximum surface coverage between 4 and 12 h after their addition, depending on the origin of the fatty acids, type of fatty acids, and proportion of fatty acids [5,8,23,25,49], once the optimal dose of inhibition was determined, potentiodynamic polarization curves were performed, at different immersion times of the working electrode, at a scan rate of 1 mV/s from −400 mV to +600 mV with respect to the corrosion potential. Each polarization curve was obtained with new working electrodes at different immersion times (0, 1, 3, 6, 9, 12, 18, and 24 h). The electrochemical parameters (Ecorr, Icorr, anodic, and cathodic slopes) were determined according to the Tafel extrapolation procedure.

## 4. Conclusions

A green dialkyl-diamide inhibitor from coffee bagasse oil was evaluated as a sweet rust inhibitor. From the OCP measurements, a rapid increase in OCP values was observed at the time of its addition, thereby causing a more noble behavior of the metal surface. LPR measurements showed a large increase in Rp values indicating that a protective film is developing on the metal surface due to the adsorption of inhibitor molecules, and that the optimal inhibition concentration is 25 ppm with an inhibition efficiency of 99%. In addition, by means of EIS measurements, the optimal concentration of inhibition was confirmed, and the Rct values obtained showed the same behavior as the Rp values. The evolution of the impedance spectra suggests that the adsorption of the inhibitor to the metal surface occurs from the diaminodiethylamine group to the unsaturations present in the hydrocarbon chains. The potentiodynamic polarization tests carried out at different immersion times have shown that when the inhibitor is added, the values of Ea and ΔH increase, but as the immersion time elapses, and with it the surface coverage, these tend to decrease to values similar to those observed in the absence of the inhibitor, in addition to the fact that the values of ΔS tend to be more negative because of the formation of a more stable and uniform protective layer on the metallic surface.

## Figures and Tables

**Table 1:** Activation parameters of the corrosion process of API-X52 steel in the absence and presence of inhibitor.

		0 ppm			25 ppm	
Time (h)	Ea (J mol^−1^)	ΔH° (J mol^−1^)	ΔS° (J mol^−1^K^−1^)	Ea (J mol^−1^)	ΔH° (J mol^−1^)	ΔS° (J mol^−1^K^−1^)
0	13,237	10,475	−284	13,237	10,475	−284
3	13,853	11,091	−285	36,304	33,542	−232
6	13,287	10,526	−288	28,422	25,660	−263
9	11,590	8828	−294	22,687	19,925	−285
12	12,938	10,176	−291	21,606	18,845	−291
18	12,672	9910	−294	17,098	14,337	−308
24	12,027	9266	−296	13,404	10,643	−323

## Data Availability

Not applicable.

## Sample Availability

Samples of the compounds are not available from the authors.
